# Effects of zacopride and multidimensional impacts of cross-kingdom symbiosis: gut microbiota modulates coronary microvascular dysfunction via the chlorophyll/heme-tryptophan metabolic axis

**DOI:** 10.1186/s12967-025-07048-3

**Published:** 2025-10-14

**Authors:** Zelin Chen, Yiding Jia, Hao Li, Rong Fan, Yuchen Cao, Lin Ni, Luqun Yang, Zitong Yuan, Kaiyi Zhu, Yuping Gao, Yuanyuan Lin

**Affiliations:** 1 Shanxi Bethune Hospital, Shanxi Academy of Medical Sciences, Tongji Shanxi Hospital, Third Hospital of Shanxi Medical University, Taiyuan, 030032 China; 2 Department of Cardiovascular Medicine, Shanxi Bethune Hospital, Shanxi Academy of Medical Sciences, Tongji Shanxi Hospital, Third Hospital of Shanxi Medical University, No. 99, Longcheng Street, Taiyuan, 030032 China; 3 Department of Cardiology, LinFen Central Hospital, LinFen, 041000 China

**Keywords:** Coronary microvascular dysfunction, Zacopride, Chlorophyll a, Tryptophan, Bacterial-fungal interactions

## Abstract

**Background:**

Coronary Microvascular Dysfunction (CMD) represents a critical pathological substrate for ischemic heart disease and is strongly associated with major adverse cardiovascular events. Zacopride, known for its dual cardiovascular regulatory properties targeting the 5-HT_4_ receptor and Kir2.1 channel, lacks evidence regarding its systemic impact on the gut microbiota-metabolism axis. Therefore, this study aims to elucidate the structural and metabolic characteristics of gut bacteria and fungi in CMD, and to explore the multidimensional therapeutic mechanisms of Zacopride through "microbial remodeling-metabolic regulation-microcirculation repair."

**Methods:**

Sixty Sprague–Dawley rats were randomized into three groups: coronary microvascular dysfunction (CMD), healthy control (NC), and Zacopride intervention (ZAC). CMD and ZAC groups received high-fat diet plus streptozotocin (STZ, 35 mg/kg) for modeling. ZAC rats were orally administered 5 mg/kg Zacopride daily for 7 days. Transthoracic Doppler echocardiography measured left anterior descending coronary artery resting/stress peak flow velocity and coronary flow reserve (CFR). Ileocecal contents underwent bacterial-fungal metagenomic sequencing to identify differential metabolic pathways. Spearman's correlation assessed cross-kingdom ecological interactions. Nine machine learning algorithms constructed classification models, with Random Forest (RF) and an optimal model identifying key genera. Linear Discriminant Analysis Effect Size validated microbial biomarkers.

**Results:**

Zacopride partially restored the CFR in CMD rats, demonstrating a therapeutic effect, and exerted a beneficial influence on the structure and diversity of the gut microbiota. The CMD state significantly reduced the expression levels of the Chlorophyll a and tryptophan metabolic pathways in the gut microbiota. Zacopride specifically restored the Chlorophyll a pathway but did not significantly recover the tryptophan metabolic pathway. RF and Elastic Net (ENET) identified *JC017*, *Chromelosporium*, and *Barnesiella* as biomarker microbiota for CMD. Notably, *JC017* primarily mediate the therapeutic effects of Zacopride via direct or indirect modulation of the Chlorophyll a metabolic pathway. *Chromelosporium*, acting as an interactive hub between fungi and bacteria, formed a cross-kingdom symbiotic relationship with *Bradyrhizobium*. Additionally, the reduction in *Barnesiella* abundance constitutes a distinctive feature of gut microbial dysbiosis in CMD.

**Conclusion:**

This study provides the first evidence that the gut microbiota modulates the pathogenesis of CMD through the "chlorophyll/heme-tryptophan metabolic axis." Furthermore, we demonstrate that Zacopride exerts therapeutic effects by remodeling microbiota-host interactions and regulating this metabolic axis, revealing a novel mechanistic link between microbial metabolism and CMD progression.

**Graphical abstract:**

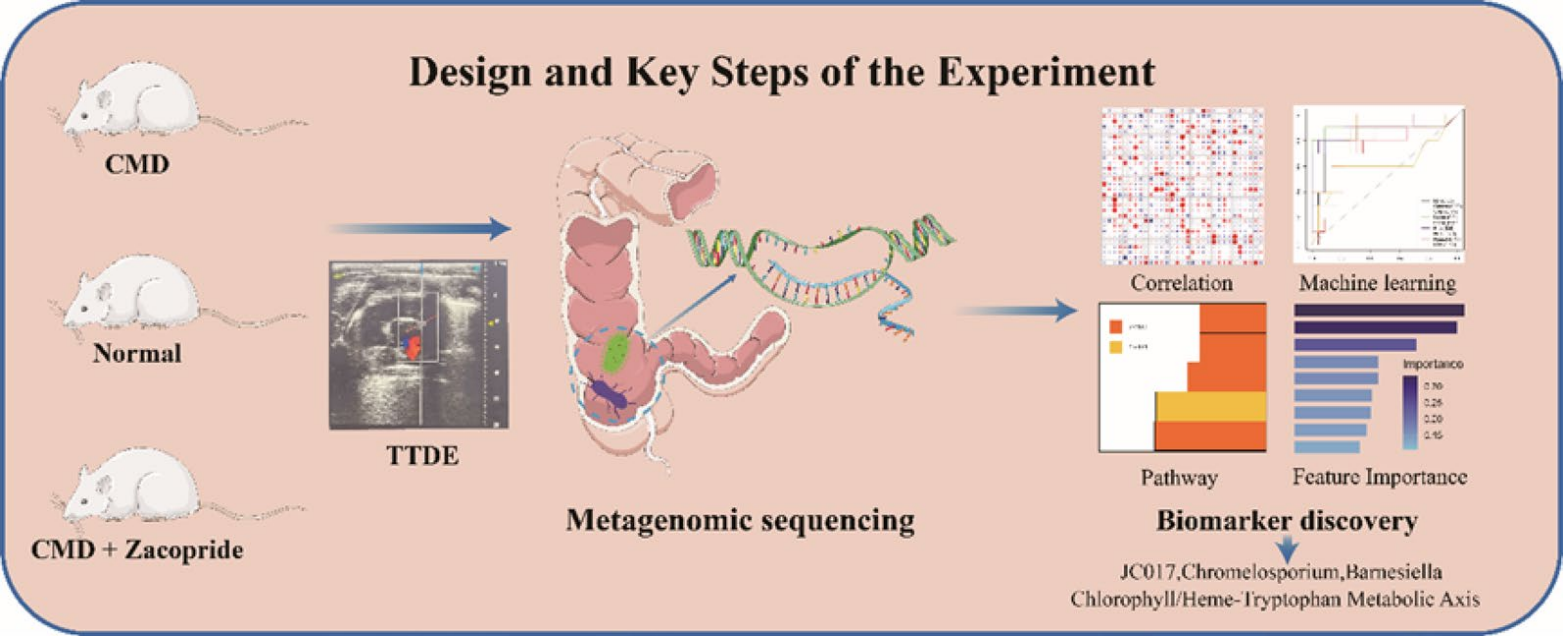

**Supplementary Information:**

The online version contains supplementary material available at 10.1186/s12967-025-07048-3.

## Introduction

Coronary microvascular dysfunction (CMD), characterized by impaired microvascular function and abnormally elevated resistance, is a critical pathological basis for ischemic heart disease. Epidemiological data indicate that approximately 50% of patients with typical angina symptoms and objective evidence of ischemia (e.g., electrocardiographic or imaging abnormalities) do not exhibit significant coronary artery obstruction [1]. CMD has been demonstrated to be significantly associated with an increased risk of major adverse cardiovascular events (MACE) and heart failure with preserved ejection fraction (HFpEF). However, due to its insidious nature, difficulties in early clinical diagnosis, and the lack of specific treatment options, in-depth research into the pathophysiological mechanisms of CMD and the identification of potential therapeutic targets are of great clinical significance. Nevertheless, traditional cardiovascular risk factors (e.g., hypertension, diabetes, and dyslipidemia) can only explain less than 20% of the risk of CMD [2], suggesting the existence of regulatory networks that remain inadequately understood in its pathological mechanisms.

Gut microbiota dysbiosis has been identified as a novel regulatory factor in cardiovascular diseases [3–7]. The human gut is colonized by over 100 trillion microorganisms, which engage in bidirectional interactions with the host through metabolites such as trimethylamine N-oxide (TMAO) and short-chain fatty acids (SCFAs). These interactions directly regulate systemic inflammatory responses, glucose and lipid metabolism homeostasis, and vascular endothelial function. In the human gut microbiota, Bacteroides, Faecalibacterium, Lactobacillus, and Bifidobacterium are four dominant bacterial genera closely associated with host health [8]. However, research on fungi remains limited [9]. Recent studies on gut microbiota increasingly employ machine learning, enabling computers to autonomously "learn" (i.e., fit training data) and generate empirical models from data, thereby uncovering deep interactions between the microbiome and host phenotypes from complex gut microbiota data [10–13]. Since the impact of microorganisms on host phenotypes is often determined not by one or two specific bacteria but by the synergistic effects of microbial communities, amplicon sequence variants (ASVs) derived from 16S rRNA gene sequence clustering can be used to construct classification and prediction models using various machine learning algorithms after data preprocessing. This approach facilitates the exploration of deep interactions between the microbiome and host phenotypes [14].

Zacopride has garnered attention due to its unique dual cardiovascular regulatory properties: (1) As a concentration-dependent 5-HT_4_ receptor agonist, it promotes L-type calcium channel phosphorylation to enhance myocardial contractility, although supratherapeutic doses may induce arrhythmias [15–17]; (2) By specifically activating the Kir2.1 inward rectifier potassium channel, it inhibits pathological calcium overload and TGF-β1/Smad-mediated myocardial fibrosis, demonstrating anti-remodeling effects in diabetic and ischemic cardiomyopathy models [18–20]. However, significant knowledge gaps remain in current research: the arrhythmogenic profile of this drug has not been fully elucidated, and its regulatory role in the α1-adrenergic pathway during stress-induced "gut-heart interactions" lacks systematic investigation [21]. Consequently, Zacopride may influence intestinal barrier function or induce intestinal inflammatory responses, further altering the composition and metabolic functions of the gut microbiota. Based on these findings, we propose a key scientific hypothesis: the therapeutic effects of Zacopride on CMD may partially stem from its systemic remodeling of the "gut microbiota-metabolism axis," thereby establishing a novel multidimensional therapeutic paradigm of "microbial remodeling-metabolic regulation-microcirculation improvement."

In microbiome research, "cross-kingdom" specifically refers to functional interactions between distinct evolutionary lineages (i.e., bacteria, fungi, and viruses) within the gut ecosystem. Currently, the microbiome field is actively advancing our understanding of such cross-kingdom interactions, which have emerged as a cutting-edge focus in microbiome research [22, 23]. This study particularly emphasizes the dynamic interplay between bacteria and fungi. From a biological perspective, bacteria and fungi belong to distinct domains of life, with fundamental differences in cellular structure, metabolic pathways, and ecological niches, making their interactions inherently "cross-kingdom" in nature.

Based on the aforementioned background, this study aims to establish a rat model of CMD and integrate coronary microcirculation functional assessment, gut microbiota metagenomic sequencing, and machine learning algorithms to elucidate the host-microbiome interaction network. By identifying key microbiota involved in the pathology of CMD and evaluating the effects of Zacopride on coronary function and gut microbiota composition in CMD rats, this research seeks to provide new theoretical insights into the "microbiota-metabolism-microcirculation" trinity mechanism of Zacopride in CMD [24, 25].

## Methods

### Animal grouping and model establishment

This experiment utilized 60 male Sprague–Dawley (SD) rats aged 8 weeks, weighing 180 ± 10 g, provided by the Animal Center of Shanxi Medical University. All animals were housed under standardized environmental conditions, with a temperature of 23 ± 2 °C, humidity of 50 ± 10%, and a 12-h light/dark cycle. Prior to the experiment, all rats were allowed free access to food and water during a 1-week acclimatization period, during which they were fed a standard diet (18% protein, 4.5% fat, 54% carbohydrates). After the acclimatization period, the rats were randomly divided into three groups: the coronary microvascular dysfunction model group (CMD group, n = 30), the healthy control group (NC group, n = 20), and the Zacopride intervention group (ZAC group, n = 10). Both the CMD and ZAC groups were fed a high-fat diet (35.5% fat, 20.6% protein, 43.9% carbohydrates), while the NC group continued on the standard diet. After 2 weeks, the CMD and ZAC groups received a single intraperitoneal injection of streptozotocin (STZ, 35 mg/kg) to establish the CMD model. A random blood glucose level ≥ 16.7 mmol/L was considered the criterion for successful CMD model establishment. Rats that did not meet this criterion received an additional injection of 30 mg/kg STZ to ensure successful model construction. The NC group was injected with an equal volume of deionized water as a control. After successful establishment of the CMD model, the ZAC group received daily oral gavage of Zacopride solution (5 mg/kg) for 7 consecutive days. At the end of the experiment, blood samples were collected from the tail vein of all rats to measure blood glucose levels, and the final body weight of each group was recorded.

### Transthoracic Doppler echocardiography (TTDE)

SD rats were anesthetized using an isoflurane gas anesthesia system (initial concentration 5%, oxygen flow rate 1.5–2.0 L/min), with continuous anesthesia maintained during the procedure (maintenance concentration 2.0–3.0%, oxygen flow rate 1.5–2.0 L/min). After being fixed in a supine position, the hair on the chest area was removed using depilatory cream, and the rats were placed on a temperature-controlled platform (37–38 °C). Heart rate was continuously monitored, and anesthesia was maintained via a nose cone. An echocardiography system was used to obtain the parasternal short-axis view (PSAX) with the assistance of a guide rail. The probe was moved rostrally to locate the pulmonary artery and then slightly shifted caudally to visualize the left anterior descending coronary artery (LAD). When individual variations made the LAD difficult to locate, the probe was adjusted to the lateral side of the pulmonary artery or the animal's body position was tilted. After recording the resting peak diastolic flow velocity of the LAD, a 25G butterfly needle was inserted into the tail vein and secured. Adenosine (140 μg·kg⁻^1^·min⁻^1^) was continuously infused at a constant rate using a microinfusion pump to induce maximal coronary hyperemia. After screening, 42 rats qualified for the study, in which resting peak flow velocity (RPFV) and stress-induced peak flow velocity (SPFV) were measured, and the mean flow velocities under both conditions were calculated to determine the coronary flow reserve (CFR).

### High-throughput sequencing and data analysis

To investigate the gut-heart axis mechanism of Zacopride in treating CMD, after completing the CFR measurement, the anesthetized rat was immediately transferred to a pre-filled, airtight euthanasia chamber containing 100% CO₂, which was continuously infused at a flow rate of 30% of the chamber volume per minute for 5 min, until complete respiratory and cardiac arrest occurred. The CO₂ supply was then terminated, followed by a 2-min observation period to confirm death. Subsequently, the ileocecal intestinal contents were aseptically collected and stored at −80 °C. Total microbial DNA was extracted using the Bead Beating method, followed by PCR amplification using bacterial-specific primers 338F/806R (targeting the V3-V4 region of the 16S rRNA gene) and fungal primers ITS_V1-F/R (targeting the ITS1 region). FASTQ data obtained from Illumina sequencing were preprocessed using QIIME2 (v2019.4). The DADA2 algorithm was employed to remove low-quality sequences and chimeras, and an ASVs feature table was constructed. Single-sequence ASVs were filtered out, and intergroup species composition analysis was performed based on the grouped ASV matrix, generating a Venn diagram. Bacterial annotation was conducted using the Greengenes database, while fungal classification was based on the UNITE database (v9.0), both achieved through 16S and ITS-specific Naive Bayes classifiers, respectively. Microbial community analysis was performed based on the ASV feature table, calculating the relative abundance of phylum- and genus-level taxonomic units and conducting principal coordinate analysis (PCoA). For functional prediction, a phylogenetic tree was constructed using PICRUSt2, and gene family copy numbers were inferred using the Castor algorithm and mapped to the KEGG database. Metabolic pathways were annotated using the MinPath method. Differential metabolic pathways were normalized, and intergroup differences were assessed using the fitFeatureModel function in R, visualized as bar plots.

### Interpretable machine learning for screening key feature species

To identify potential biomarkers and therapeutic target microbiota, this study systematically constructed classification prediction models based on nine classic machine learning algorithms at the genus level for the CMD group versus the NC group and the CMD group versus the ZAC group. The algorithms included Random Forest (RF), Gradient Boosting, Support Vector Machine (SVM), Lasso Regression/L1 Regularization, Ridge Regression/L2 Regularization, Elastic Net (ENET), k-Nearest Neighbors (kNN), Neural Network, and Linear Discriminant Analysis (LDA). For each machine learning algorithm, 100 independent repeated experiments were conducted. In each experiment, the dataset was randomly divided into five subsets (fivefold cross-validation), with one subset sequentially selected as the validation set and the remaining four subsets as the training set. Model performance was evaluated using the Receiver Operating Characteristic (ROC) curve and the area under the curve (AUC). The performance of each experiment was determined by the average of the fivefold cross-validation results, and the final model performance was assessed by calculating the mean AUC across 100 experiments. The classic RF model and another top-performing model were selected to interpret their feature importance. Additionally, Linear Discriminant Analysis Effect Size (LEfSe) was used to validate the identified differential species, The significance threshold was set at an LDA score > 2, and only species with significant differences were retained. The results were visualized using cladograms.

### Statistical methods

All statistical analyses and visualizations were performed using R statistical software (version 4.2.0). The data analysis strategy followed these steps: First, the Kruskal–Wallis one-way non-parametric rank-sum test was used to globally assess the overall median differences among the three groups. Upon obtaining significant results, Dunn's multiple comparison test was further applied for pairwise group comparisons, with the Benjamini–Hochberg method used to correct for multiple testing errors. To evaluate the ecological interactions between bacterial and fungal communities, Spearman's rank correlation analysis was employed for non-parametric correlation assessment. The association network was visualized using heatmaps and Cross-kingdom Co-occurrence Networks, with significance levels (*p* < 0.05, *p* < 0.01, *p* < 0.001) annotated to indicate the degree of significance.

## Result

### Basic characteristics and CFR of rats

**Fig. 1:**
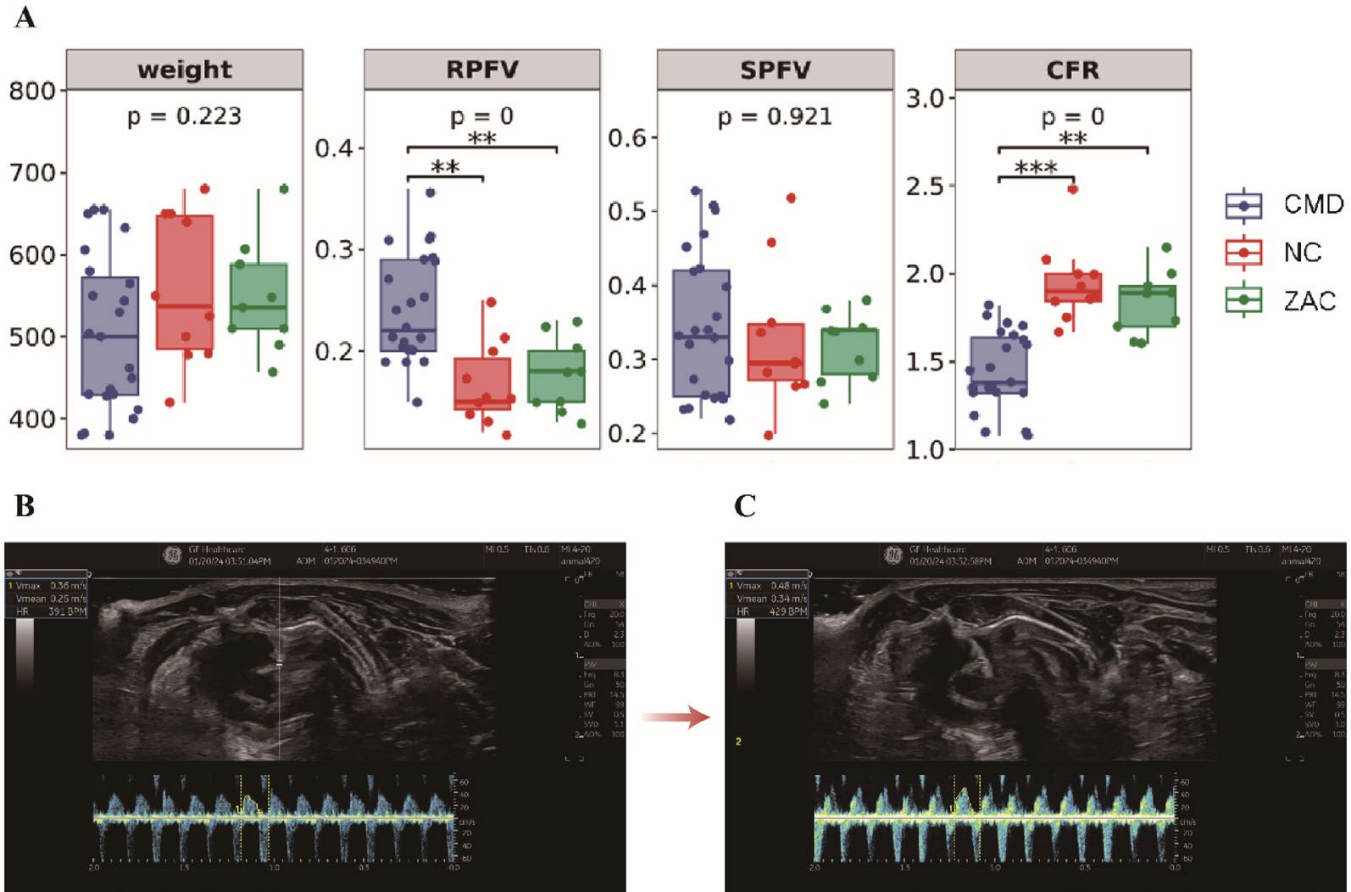
**A** Bodyweight and coronary flow reserve (CFR) in experimental groups: coronary microvascular dysfunction (CMD, n = 23), normal controls (NC, n = 10), Zacopride-treated CMD (ZAC, n = 9). **B**, **C** Representative Doppler Echocardiography images of coronary artery blood flow in CMD rats before (Baseline) and after (Post-adenosine) adenosine stress testing. Intergroup differences were analyzed using the Kruskal–Wallis test, followed by Dunn's multiple comparisons test if significance was reached (** *p* < 0.01, *** *p* < 0.001). RPFV, resting peak flow velocity; SPFV, stress-induced peak flow velocity

In this study, TTDE was used to measure coronary artery flow velocity waveforms in rats, and CFR was employed to quantitatively evaluate coronary microcirculation function. The bar chart illustrating intergroup differences (Fig. 1) showed that the CMD group had the lowest overall body weight trend (*p* > 0.05) and exhibited symptoms such as dull fur, poor mental state, and heightened stress response. Additionally, both RPFV and SPFV levels in the CMD group were significantly higher compared to the NC and ZAC groups (*p* < 0.01). Although the CMD group demonstrated higher baseline and stress-induced peak flow velocities, its CFR was significantly lower than that of the NC group (*p* < 0.001) and the ZAC group (*p* < 0.01), indicating that CMD rats had higher coronary artery flow velocities but relatively impaired coronary microcirculation function. We hypothesize that this is due to hemodynamic changes caused by coronary atherosclerosis, which may further adversely affect shear stress, axial plaque stress, and plaque stability. In conclusion, Zacopride indeed partially restored the CFR in CMD rats, demonstrating a therapeutic effect.

### Diversity indices of bacteria and fungi

**Fig. 2:**
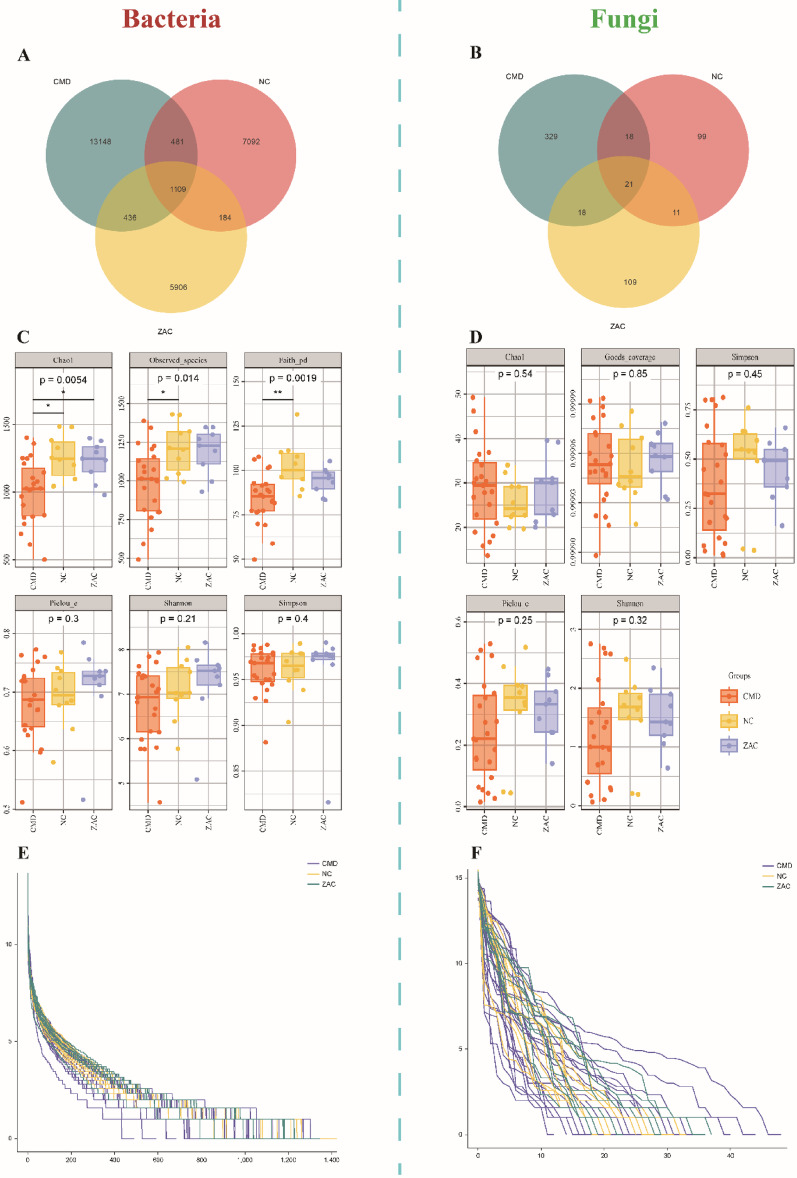
A and B delineate the compositional heterogeneity of amplicon sequence variants (ASVs) for bacterial (**A**) and fungal (**B**) communities through Venn diagram analysis. **C** and **D** quantitatively depict the statistical differences in α-diversity indices between groups for bacterial (**C**) and fungal (**D**) communities, respectively, using bar charts. **E** and **F** systematically illustrate the structural characteristics of species abundance rank distributions for bacterial (**E**) and fungal (**F**) communities, respectively, via rank-abundance curves. The bacterial richness and diversity in the Coronary Microvascular Dysfunction (CMD, n = 23 rats) group were significantly lower than those in the healthy control (NC, n = 10 rats) group, while the Zacopride intervention (ZAC, n = 9 rats) group partially restored bacterial abundance. Although fungal diversity showed no significant differences among groups, a trend of microbial dysbiosis was still observed. The microbial diversity in the ZAC group was closer to that of the NC group, indicating a certain therapeutic effect. Intergroup differences were analyzed using the Kruskal–Wallis test, followed by Dunn's multiple comparisons test if significance was reached (* *p* < 0.05, ** *p* < 0.01)

To explore the shared and unique microbial distributions among the three groups, we analyzed the microbial communities using Venn diagrams. By comparing the ASVs across groups, we quantified the number of members in different sets and the shared ASVs among groups. The results of bacterial diversity differences among the CMD, NC, and ZAC groups indicated that, under sufficient sequencing depth, the richness and diversity of the bacterial community in the CMD group were significantly reduced (Fig. 2). The Chao1 index and Observed species values were the lowest in the CMD group, suggesting that species abundance was suppressed by the disease state (*p* < 0.05) [26]. Faith's pd index further revealed that the CMD group had the lowest microbial phylogenetic diversity (*p* < 0.01), indicating a significant reduction in the evolutionary breadth of its microbial populations [27]. The ZAC group partially reversed this trend, with significant increases in the Chao1 and Observed species values compared to the CMD group (*p* < 0.05), suggesting that Zacopride may improve metabolic disorders by modulating microbial abundance. Diversity analysis further showed that the CMD group had the lowest Pielou evenness and Shannon diversity indices (*p* < 0.05) and the highest Simpson index (*p* < 0.01), indicating significant microbial dysbiosis in this group. For fungal diversity, no significant differences were observed between the CMD and NC groups (Chao1 richness index and Shannon diversity index, both *p* > 0.05), but a trend of microbial dysbiosis was noted. The ZAC group also did not significantly alter the community structure: its Chao1 index showed no significant differences compared to the NC and CMD groups (both *p* > 0.05), and the Shannon and Pielou evenness indices did not reach statistical significance (both *p* > 0.05). The Simpson index indicated minor changes in community dominance between the ZAC and NC groups, but the fungal diversity in the ZAC group was closer to that of the NC group, suggesting that Zacopride partially altered fungal α-diversity, although its restorative effect was less pronounced compared to that on bacteria.

### Species composition analysis

**Fig. 3:**
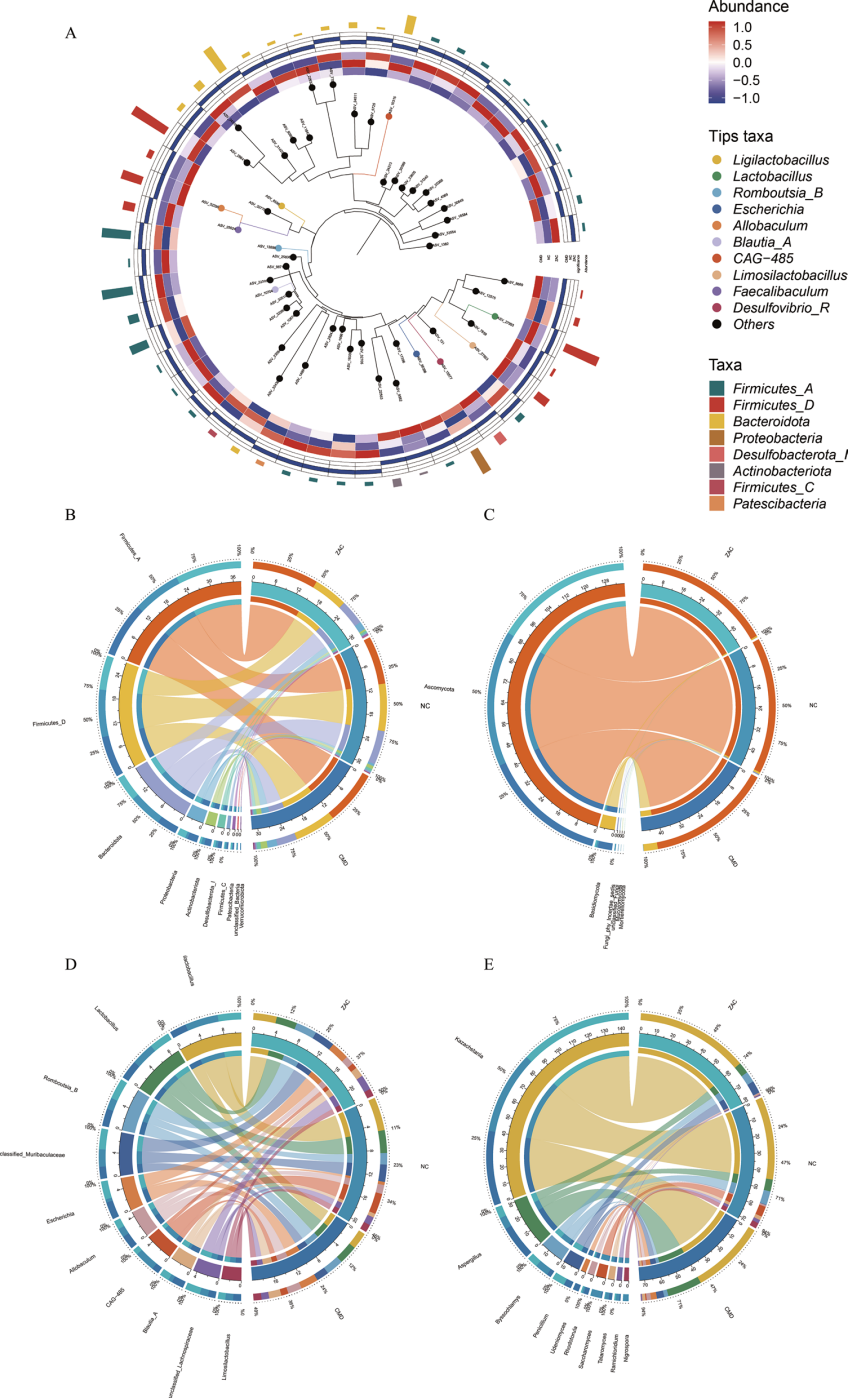
(**A**) phylogenetic tree (PhyloTree) evolutionary tree of bacteria, integrating species abundance, evolutionary position, and genetic distance information. (**B**, **C**) Circos plots illustrating the relationships of bacterial (**B**) and fungal (**C**) communities at the phylum level, showcasing the structural characteristics of phylum-level taxonomic units. (**D**, **E**) Circos plots depicting the interaction networks of bacterial (**D**) and fungal (**E**) communities at the genus level, revealing the structural characteristics of genus-level taxonomic units. Coronary Microvascular Dysfunction (CMD) significantly altered the community structure of gut bacteria and fungi, indicating a close association with gut microecological imbalance; meanwhile, Zacopride restored the abundance of beneficial bacteria and modulated the microbial community structure, providing potential evidence for alleviating CMD. Experimental groups: CMD (n = 23), healthy control (NC, n = 10), and Zacopride intervention (ZAC, n = 9)

We investigated the structural changes in the gut microbiota of the CMD, NC, and ZAC groups through bacterial phylogenetic tree (PhyloTree) and taxonomic composition analysis (Fig. 3). The results revealed significant alterations in the bacterial community structure in the CMD group, particularly a notable increase in the abundance of *Firmicutes* and *Proteobacteria*, especially the genera *Enterobacter* and *Escherichia-Shigella*. This suggests that CMD may lead to gut microbiota dysbiosis and trigger inflammatory responses. Concurrently, the abundance of beneficial bacteria such as *Bacteroidetes* and *Lactobacillus* significantly decreased in the CMD group, suggesting that the reduction of beneficial gut bacteria may be closely related to the onset and progression of coronary microvascular dysfunction. In contrast, the bacterial community structure in the NC group remained relatively stable, with *Firmicutes* and *Bacteroidetes* as the dominant phyla, and higher abundances of beneficial bacteria such as *Lactobacillus*, *Bifidobacterium*, and *Faecalibacterium*, maintaining a healthy gut microecological state. In the ZAC group, we observed that Zacopride intervention promoted an increase in the abundance of *Bacteroides* and *Faecalibacterium*, suggesting that Zacopride aids in the restoration of gut health by modulating the gut microbiota structure. Additionally, the abundance of some *Proteobacteria* bacteria slightly decreased in the ZAC group, suggesting that the drug may suppress inflammation induced by microcirculatory dysfunction.

In terms of fungal community structure, analyses at both the phylum and genus levels revealed certain differences. At the phylum level, the abundance of *Ascomycota* in the CMD group was significantly lower than that in the NC group, while the abundance of *Basidiomycota* significantly increased. After Zacopride treatment, the abundance of *Ascomycota* recovered to levels close to those of the NC group, and the abundance of *Basidiomycota* decreased, suggesting that the drug partially reversed the dysbiosis of fungal communities at the phylum level. At the genus level, the CMD group exhibited enrichment of potentially pathogenic fungi and a reduction in symbiotic fungi: the genera *Aspergillus* and *Rhodotorula* significantly proliferated, while the abundance of *Saccharomyces* and *Byssochlamys* significantly decreased. This suggests that the increase in pathogenic microorganisms and the decrease in beneficial fungi in the CMD group may be associated with pathological changes. After Zacopride intervention, the abundance of the pathogenic fungus *Aspergillus* significantly decreased, while the abundance of *Kazachstania* markedly increased, surpassing that of the CMD group. However, the restorative effects of Zacopride on *Saccharomyces* and *Nigrospora* were limited. Notably, the abundance of *Penicillium* in the ZAC group abnormally increased to 8.33% (NC group: 0.25%), a phenomenon that may require further evaluation of the drug's clinical safety considering its toxin-producing potential. Overall, Zacopride gavage treatment improved gut microecological balance, restored the abundance of beneficial microbiota, and alleviated the negative impact of coronary microvascular dysfunction on the structure of the gut microbiota.

### β-Diversity of gut microbiota

**Fig. 4:**
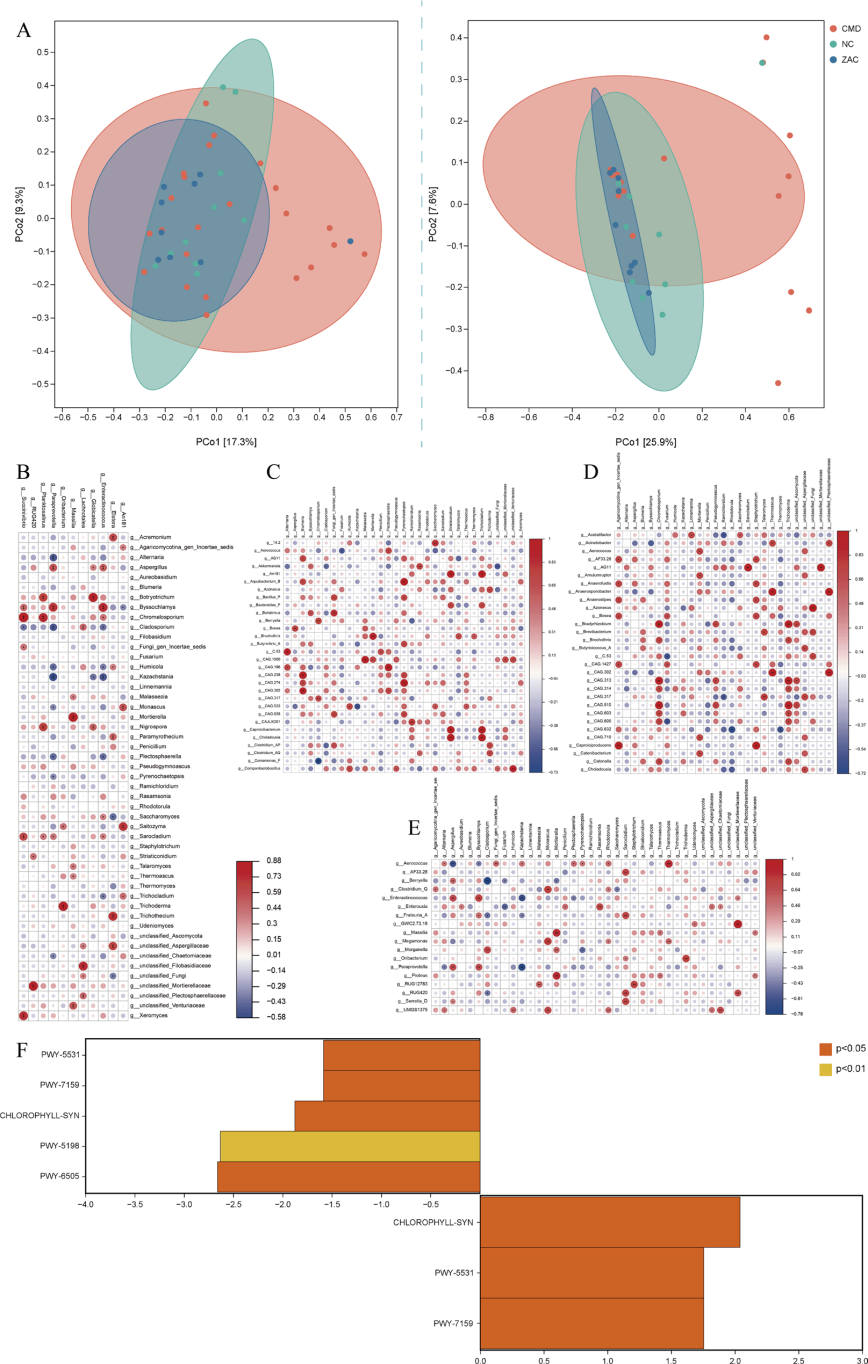
**A** Principal coordinates analysis (PCoA) illustrates the differences in bacterial (left) and fungal (right) communities. The Coronary Microvascular Dysfunction (CMD, n = 23 rats) group exhibits a scattered distribution, indicating reduced microbial community stability, while The Zacopride intervention (ZAC, n = 9 rats) group exhibits an intermediate distribution between the CMD and healthy control (NC, n = 10 rats) groups, suggesting that Zacopride partially restored microbial stability. **B**, **C**, **D**, **E** Heatmaps display significant genus-level correlations between bacteria and fungi under four conditions: overall sample environment (n = 42 rats), CMD, healthy state, and ZAC intervention. Ecological interactions between bacterial and fungal communities were assessed using Spearman's rank correlation analysis, with significantly correlated taxa pairs visualized via network diagrams (* *p* < 0.01, ** *p* < 0.001). Notably, Chromelosporium demonstrates significant correlations with bacteria across multiple ecological contexts, particularly under ZAC intervention, where it forms a highly synergistic cross-kingdom symbiotic relationship with Bradyrhizobium, serving as a key hub for maintaining gut microbiota stability and functionality. (F): The left panel highlights metabolic pathways significantly downregulated in the CMD group, primarily including Chlorophyll a synthesis and tryptophan degradation pathways. The right panel shows metabolic pathways significantly upregulated in the ZAC group, indicating that Zacopride selectively modulates the Chlorophyll metabolic network in gut microbiota, thereby ameliorating CMD-related metabolic disorders. Differential pathways were identified using metagenomeSeq's zero-inflated log-normal model with false discovery rate (FDR) correction (*p* < 0.05)

Based on the distance matrix between samples, the PCoA algorithm effectively captured and retained key information on species differences for each sample (Fig. 4A), reflecting the β-diversity of the gut microbiota. The results showed that the samples in the NC group were more clustered on the plot, indicating smaller intra-group differences and relatively stable microbiota. By contrast, the CMD group exhibited a more scattered distribution across both dimensions, indicating significantly increased intra-group variability and a more complex and unstable gut microbial community. The distribution of the ZAC group fell between that of the CMD and NC groups, indicating that Zacopride treatment partially improved the state of the gut microbiota. Overall, the CMD condition led to impaired diversity and phylogenetic complexity of the gut microbiota, while ZAC treatment partially restored microbial stability and diversity, thereby ameliorating related metabolic disorders.

### Overall mean correlation analysis of bacterial-fungal genus interactions

Through cross-kingdom microbial interaction network analysis, we systematically examined the interaction patterns between bacterial and fungal genera. Correlation analysis based on overall means revealed multiple significant positive relationships between bacterial and fungal genera (Fig. 4B). Among these, the association between *Succinivibrio* and *Chromelosporium* was the most significant (*r* = 0.879, *p* < 0.001), followed by *Oribacterium* and *Trichoderma* (*r* = 0.806, *p* < 0.001). These microbial groups may serve as stable cross-kingdom functional modules, providing critical references for health status, while also aiding in identifying changes under CMD and Zacopride intervention.

### Cross-kingdom interactions of bacteria and fungi in the CMD group

In the CMD group, cross-kingdom interactions within the microbial community exhibited distinctive pathological association patterns (Fig. 4C). The study revealed a highly positive correlation between *Aerococcus* and *Mortierella* (*r* = 0.850, *p* < 0.001). Additionally, both *Massilia* and *Proteus* showed significant interactions with the thermophilic fungal genus *Thermomyces* (*r* = 0.835, *p* < 0.001). Most notably, an unclassified member of *Mortierellaceae* exhibited a perfect positive correlation with the *GWC2.73.18* (*r* = 1.000, *p* < 0.001), suggesting that they may share highly consistent functional or distributional characteristics in the pathological process of CMD. These findings provide insights into the complex interactions of microbial communities in the pathogenesis of CMD.

### Cross-kingdom interactions of bacteria and fungi in the healthy control group

In the healthy control group, the core symbiotic network of the microbial community exhibited distinct characteristics (Fig. 4D). *CAG-1000*, *Massilia*, *Mesorhizobium_F*, and *Succinispira* all showed perfect positive correlations with the fungal genus *Malassezia* (*r* = 1.000, *p* < 0.001). Similarly, *Eisenbergiella*, *QALR01*, and *SFLA01* formed a completely positively correlated module with the fungal genus *Humicola* (*r* = 1.000, *p* < 0.001). Additionally, bacterial genera such as *Caproicibacterium*, *Choladousia*, and *Gracilibacter* exhibited complete interactions with the fungal genera *Striaticonidium* and *Trichocladium* (*r* = 1.000, *p* < 0.001). Notably, a strong association was also observed between *Enterococcus_H* and *Chromelosporium* (*r* = 0.84, *p* < 0.001). These findings suggest that these cross-kingdom combinations may constitute functional modules essential for maintaining intestinal homeostasis.

### Impact of zacopride treatment on bacterial-fungal interaction networks

Zacopride intervention significantly reshaped the cross-kingdom microbial interaction network (Fig. 4E). A perfect positive correlation was observed between the bacterial genus *AG11* and the fungal genus *Sarocladium* (*r* = 1.000, *p* < 0.001), suggesting that they may form a tightly coordinated synergy during the intervention. Additionally, *Bradyrhizobium* established complete interactions with *Chromelosporium* and *Trichoderma* (*r* = 1.000, *p* < 0.001). These multiple interaction patterns indicate that Zacopride not only increased the abundance of beneficial bacteria but also reshaped microbial interactions by enhancing cross-kingdom metabolic complementarity, thereby influencing the overall functionality of the microbial community.

**Fig. 5:**
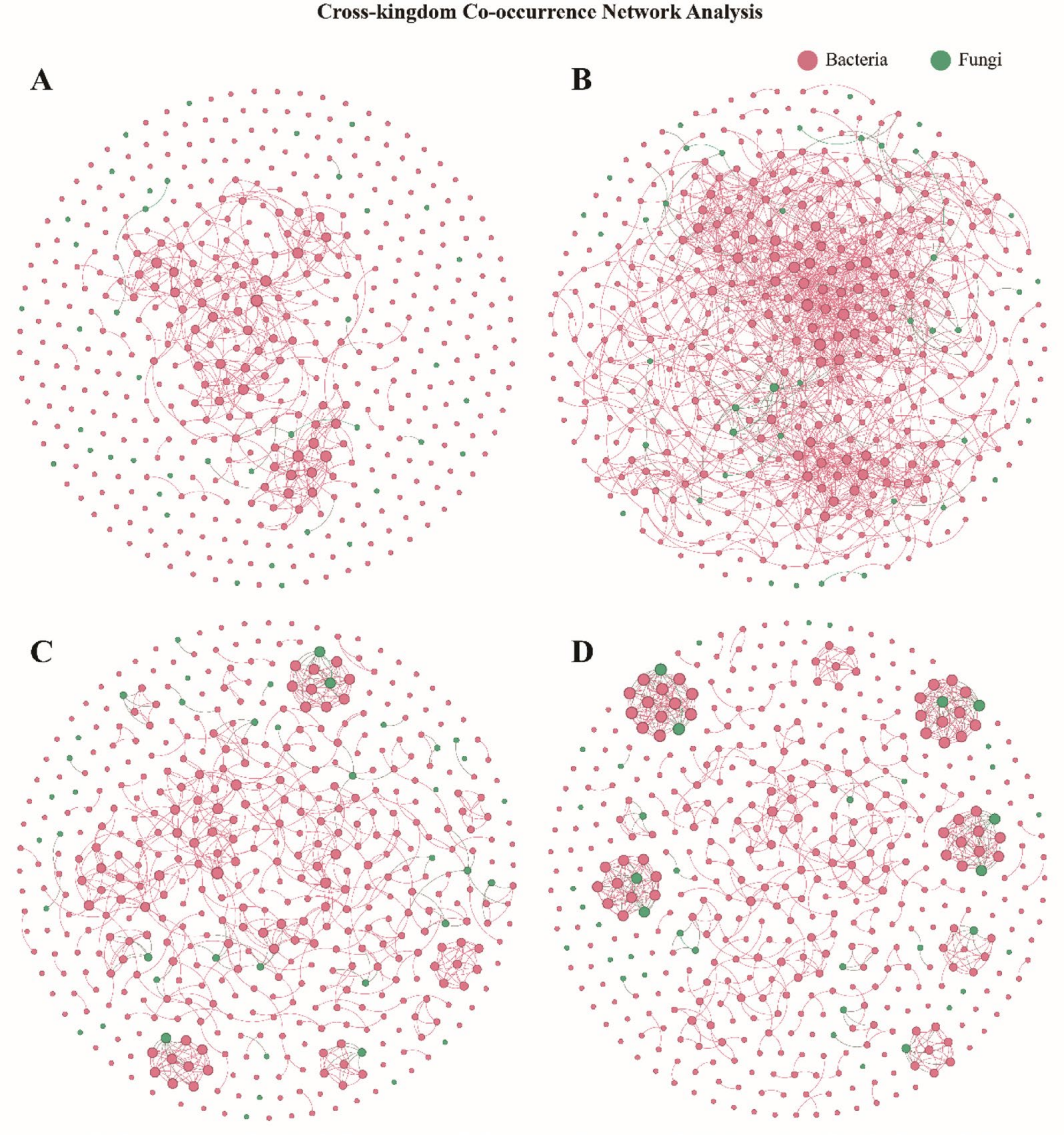
**A**, **B**, **C**, and **D** represent the Cross-kingdom Co-occurrence Networks for the overall sample environment (n = 42 rats), the Coronary Microvascular Dysfunction (CMD, n = 23 rats) group, the healthy control (NC, n = 10 rats) group, and the Zacopride intervention (ZAC, n = 9 rats) group, respectively. In each ecological context, bacteria and fungi exhibit intricate interaction networks. Notably, in the ZAC group, the Zacopride intervention significantly facilitated the formation of functional modules involving interactions between bacteria and fungi. Cross-kingdom co-occurrence networks were constructed using Spearman's rank correlation (|*r*|> 0.6, *p* < 0.001)

Notably, *Chromelosporium* was found to participate in bacterial-fungal symbiotic networks across various ecological contexts, including the overall sample environment, healthy gut conditions, and Zacopride intervention. Co-occurrence network analysis of bacterial-fungal interactions revealed that the Zacopride-treated gut environment facilitated the formation of multiple bacterial-fungal functional modules (Fig. 5). Specifically, in the gut environment under Zacopride treatment, *Chromelosporium* exhibited a highly correlated conditional symbiosis with *Bradyrhizobium*, highlighting its role as a key hub species that helps maintain the stability and functionality of the gut microbiota. Conversely, the CMD state disrupted this symbiotic network and interfered with microbial metabolism, promoting further disease progression.

### Metabolic pathway analysis in CMD rats

Compared to the NC group, CMD rats exhibited significant dysregulation of intestinal bacterial metabolic pathways, while no significant differences were observed in fungal metabolic pathways (Fig. 4F). Differential analysis revealed that five metabolic pathways showed a significant downregulation trend (*p* < 0.05), with distribution characteristics of clear biological significance: 1) Among the pathways related to Chlorophyll a biosynthesis, the aerobic light-dependent biosynthesis I pathway (CHLOROPHYLL-SYN) was the most significantly downregulated, followed by the anaerobic biosynthesis II pathway (PWY-5531) and the aerobic light-independent biosynthesis III pathway (PWY-7159). 2) In the coenzyme metabolism-related pathways, the coenzyme F420 biosynthesis pathway (PWY-5198) and the L-tryptophan degradation XII pathway (PWY-6505), which is associated with amino acid metabolism, also showed significant inhibition. This systemic metabolic disorder suggests that CMD rats may experience multidimensional pathological changes, including imbalances in the chlorophyll metabolic network, defects in coenzyme synthesis, and impairments in amino acid catabolism. Notably, Zacopride intervention demonstrated a significant selective regulatory effect on metabolic pathways. After gavage treatment, the expression levels of the three major Chlorophyll a biosynthesis pathways were significantly restored. However, the coenzyme F420 biosynthesis pathway (PWY-5198) and the L-tryptophan degradation pathway (PWY-6505) did not show statistically significant changes post-intervention. In summary, CMD primarily affects the metabolic capacity of intestinal bacteria, and Zacopride's regulation of intestinal microbiota metabolism in CMD rats is pathway-selective, primarily acting on intestinal bacteria rather than fungi, and is closely related to the restoration of energy supply disorders in the chlorophyll metabolic network.

#### Machine learning combined with LEfSe analysis to identify key microbial biomarkers

**Fig. 6:**
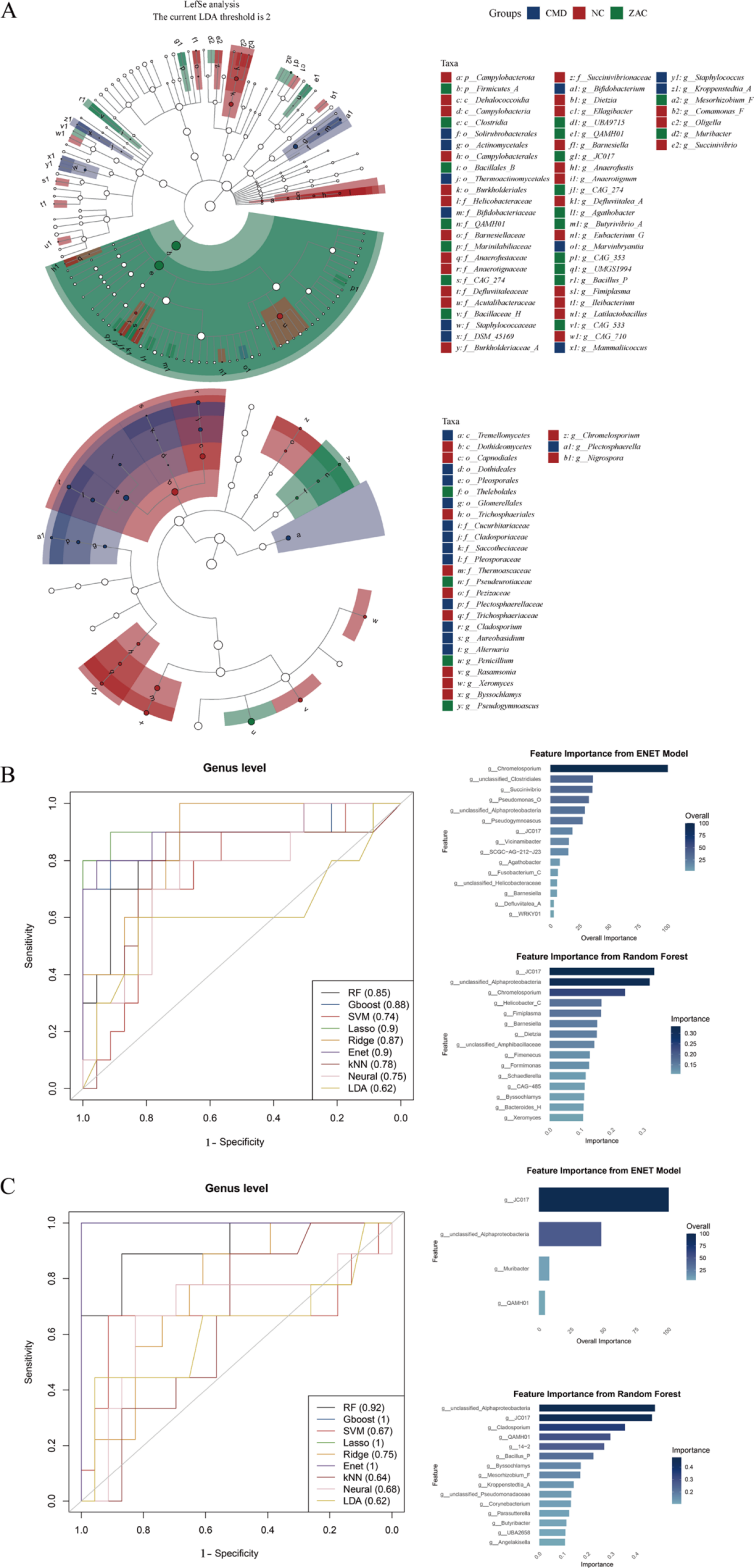
**A** The cladograms from Linear Discriminant Analysis Effect Size (LEfSe) analysis illustrate the significantly differential taxa of bacteria (top) and fungi (bottom) among the groups, revealing key microbial features associated with the Coronary Microvascular Dysfunction (CMD, n = 23 rats), healthy control (NC, n = 10 rats), and Zacopride intervention (ZAC, n = 9 rats) groups. **B**, **C** The left panels show the area under the curve (AUC) performance comparison of nine machine learning models—Random Forest (RF), Gradient Boosting (GBoost), Support Vector Machine (SVM), Lasso Regression, Ridge Regression, Elastic Net (ENET), k-Nearest Neighbors (kNN), Neural Network (Neural), and Linear Discriminant Analysis (LDA)—in genus-level classification for the CMD-NC model (**B**) and the CMD-ZAC model (**C**). The left panels display the AUC values of each model, with ENET outperforming the others in both comparisons. The right panels show the feature importance plots from the RF and ENET models, identifying overlapping core predictive genera, including JC017, Chromelosporium, and Barnesiella. These genera were further validated as differential taxa by LEfSe analysis, demonstrating their potential as robust microbial biomarkers (LDA score > 2.0, *p* < 0.05). Notably, JC017 not only plays a protective role in CMD progression but also serves a critical function in the therapeutic effects of Zacopride, highlighting its importance in disease progression and intervention

To establish a specific microbial biomarker system, this study first constructed nine machine learning models at the genus level to compare the CMD group with the NC group and the CMD group with the ZAC group. The optimal model was selected based on the AUC as the evaluation metric, and the important features of the model were interpreted. Subsequently, LEfSe analysis was employed to identify differential microbial taxa from the phylum to genus level for auxiliary validation (LDA score > 2.0, *p* < 0.05). The ENET model demonstrated superior performance in both comparative analyses, and thus, the RF and ENET models were selected for feature importance analysis (Fig. 6). The results revealed a significant overlap in the feature importance rankings of the CMD-NC model, including *Chromelosporium*, *JC017*, and *Barnesiella*. For the CMD-ZAC model, *JC017* was again identified as an important feature and confirmed as a differential microbial taxon in the ZAC group through LEfSe analysis. This suggests that *JC017* not only exerts a protective effect during CMD progression but also has the potential to serve as a specific microbial biomarker for CMD. Furthermore, it acts as a target in the therapeutic effects of Zacopride, playing a pivotal role in the onset, progression, and intervention of the disease. Additionally, *Chromelosporium*, identified as a hub fungus in cross-kingdom interaction analysis, was recognized as an important feature in the CMD-NC model and distinguished as a differential fungus in the NC group by LEfSe. This indicates its primary role in exerting protective effects during CMD progression rather than serving as a target microbial taxon for Zacopride treatment. Although the importance scoring systems of the two models differ (the RF model is based on the contribution of features to prediction outcomes, while ENET relies on standardized regression coefficients), the aforementioned overlapping features were consistently identified as core predictors in both models and confirmed as differential taxa by LEfSe analysis, suggesting their robustness as microbial biomarkers.

## Discussion

### The chlorophyll/heme-tryptophan metabolic axis influences CMD disease progression

The pathological role of tryptophan (Trp) metabolism dysregulation in cardiovascular diseases has garnered increasing attention. However, research on the association between tryptophan metabolism and CMD remains limited. Our findings suggest that the CMD state significantly reduces the expression levels of Chlorophyll a and tryptophan metabolic pathways in the gut microbiota. It is known that a bidirectional regulatory network exists between tryptophan and heme/chlorophyll metabolism, mediated by enzyme activity modulation, cofactor dependency, and metabolite interactions. This disruption in chlorophyll metabolism may affect heme synthesis through the shared glutamate precursor pool, while also influencing tryptophan metabolism via feedback regulation of porphyrin ring metabolism.

We begin by discussing the shared regulatory mechanisms between heme and chlorophyll to elaborate on the chlorophyll/heme-tryptophan metabolic network, as both pathways initiate from 5-aminolevulinic acid. Through the catalysis of different enzymes, Fe^2^⁺ or Mg^2^⁺ is inserted, ultimately forming heme or chlorophyll, respectively. CMD leads to the downregulation of Chlorophyll a biosynthesis-related pathways in the gut microbiota, which evidently synchronously affects heme synthesis. Impaired heme synthesis results in porphyrin accumulation, associated with oxidative stress, lipid peroxidation, and mitochondrial dysfunction, thereby influencing tryptophan metabolism [28]. Notably, a bidirectional regulatory network exists between tryptophan and heme: on one hand, tryptophan enhances heme biosynthesis by promoting precursor pathways; on the other hand, heme acts as an essential cofactor for tryptophan 2,3-dioxygenase (TDO) and indoleamine 2,3-dioxygenase (IDO), thereby controlling the flux of tryptophan into the kynurenine (KYN) metabolic pathway. When intracellular tryptophan levels rise, they can inhibit 5-aminolevulinic acid synthase (5-ALAS), the rate-limiting enzyme in heme synthesis, reducing heme biosynthesis and forming a negative feedback regulatory loop [29]. Inflammation and high-fat diets may alter the gut microbiota, exacerbating the metabolic imbalance between tryptophan and heme. This imbalance may further influence the onset and progression of CMD.

Tryptophan is known to exert its biological functions through three major metabolic pathways: the serotonin (5-HT) pathway, the indole metabolism pathway, and the kynurenine pathway, with the gut microbiota influencing all three processes [30]. Tryptophan metabolites, such as 5-HT, indolepropionic acid (IPA), and indole-3-acetic acid (IA), play crucial roles in regulating intestinal barrier function. Early studies have shown that IA significantly alleviates intestinal inflammation in mice by upregulating the production of the anti-inflammatory cytokine IL-10 and the expression of mucin genes, while also improving intestinal barrier function [31]. Recent research further reveals that IA, in synergy with gut bacteria, not only repairs the intestinal barrier but also significantly mitigates insulin resistance (IR) in STZ-induced type 2 diabetic rats. Additionally, IA promotes the expression of genes related to barrier integrity (such as Occludin, Claudin-1, ZO-1, and MUC2) by activating the aryl hydrocarbon receptor (Ahr) in epithelial cells, thereby restoring the intestinal barrier [32].

In addition to influencing intestinal barrier function and gut microbiota metabolism, decreased plasma tryptophan levels are also directly correlated with an increased risk of cardiovascular diseases, particularly in coronary heart disease, atherosclerosis, and myocardial infarction [33, 34]. An elevated KYN/Trp ratio has also been recognized as a predictor of cardiovascular diseases [35, 36]. Furthermore, tryptophan metabolites significantly impact lipid metabolism [37]. Studies have found that kynurenine is closely associated with triglyceride levels and the risk of atherosclerosis [38]. Additionally, the tryptophan metabolic pathway is closely linked to metabolic diseases such as diabetes, insulin resistance, and obesity. In the KYN pathway, the IDO enzyme alleviates insulin resistance by reducing inflammatory responses, which may mitigate complications of type 2 diabetes [39]. In the 5-HT pathway of tryptophan, tryptophan metabolites can influence the onset and progression of CMD by regulating insulin secretion and promoting the proliferation of pancreatic β-cells. As a concentration-dependent 5-HT receptor agonist, Zacopride's therapeutic effects on CMD may partially stem from this mechanism. The aforementioned improvements in CMD risk factors suggest that targeting the gut microbiota-heme/chlorophyll-tryptophan metabolic network could provide a novel direction for CMD treatment.

### JC017 as a key microbe in CMD disease progression and zacopride therapeutic effects

To avoid solely relying on conventional microbial diversity and structural changes to explain the "gut-heart axis" pathological mechanisms of CMD and to increase attention on fungi, this study established interpretable machine learning models using bacterial-fungal microbiome data. The results indicate that *JC017* was consistently identified as an important feature in both the CMD-NC and CMD-ZAC models, with particularly high feature contribution in the CMD-ZAC model. As a relatively understudied bacterium of the *Marinilabiliaceae* family, *JC017* is known to be an efficient producer of riboflavin [40]. We further observed that changes in the abundance of *JC017* were highly synchronized with the expression levels of the Chlorophyll a metabolic pathway. However, existing studies have not yet identified its direct impact on Chlorophyll a-related metabolic pathways.

Therefore, we propose the hypothesis that *JC017* may influence Chlorophyll a-related pathways through direct or indirect effects and further exert a protective role in CMD by modulating the chlorophyll/heme-tryptophan metabolic network, which is closely related to the pharmacological mechanism of Zacopride in regulating gut microbiota for CMD treatment. However, the impact of Zacopride on gut microbiota is complex. For instance, its sustained inhibition of *Saccharomyces* may affect tryptophan metabolism, potentially explaining why the tryptophan metabolic pathway was not significantly restored in the ZAC group, while only the expression of the Chlorophyll a pathway improved. Additionally, the increased abundance of *Penicillium* in the ZAC group may disrupt the already complex pathological intestinal microenvironment, leading to the suppression of some beneficial bacteria and masking partial therapeutic effects.

In summary, *JC017* may play a dual role in both the progression of CMD and the mechanisms of pharmacological treatment. Its consistent presence in disease progression models and treatment response profiles suggests that this bacterial strain could serve as a microbial biomarker for CMD and also act as a key target microbiota for Zacopride gavage therapy.

### Zacopride promotes the Chromelosporium-Bradyrhizobium cross-kingdom symbiosis to maintain intestinal homeostasis

*Chromelosporium* was identified as an important feature in the CMD-NC classification model and is a relatively understudied fungus belonging to the *Ascomycota* phylum. Further bacterial-fungal correlation analysis revealed that *Chromelosporium* participates in the complex correlation-based symbiotic network of the gut microbiota across the overall sample, NC group, and ZAC group. This indicates its role as an interaction hub between intestinal fungi and bacteria. Notably, a significant correlation was observed between *Chromelosporium* and *Bradyrhizobium* in the ZAC group, suggesting that Zacopride may promote a conditional symbiotic relationship between the two.

*Bradyrhizobium*, a key member of the *Pseudomonadota* phylum, has long been studied for its symbiotic nitrogen-fixing relationship with leguminous plants. However, recent clinical studies suggest that *Bradyrhizobium* may transcend its traditional ecological niche and exhibit potential pathogenicity under conditions of host immune imbalance or intestinal barrier damage. For example, *Bradyrhizobium* has been detected in the plasma microbiota of patients with liver cirrhosis, indicating a risk of systemic infection in immunocompromised states [41, 42]. In our study, however, it formed a symbiotic relationship with *Chromelosporium* in the CMD environment under Zacopride intervention, becoming one of the beneficial factors in delaying CMD progression and protecting intestinal microecological homeostasis.

Furthermore, the biological characteristics of *Bradyrhizobium* are far more complex than traditionally understood. Genomic studies reveal that this genus not only encompasses metabolic pathways centered on nitrogen fixation but also exhibits potential for photoautotrophy. Through the photosynthetic gene cluster (PGC), it encodes key enzymes for chlorophyll synthesis, further regulating the chlorophyll/heme-tryptophan metabolic network [43]. Additionally, recent studies have demonstrated that chlorophyll and its derivatives possess significant anti-genotoxic, anti-tumor, and antioxidant properties. They can function as antioxidants by modulating the metabolic homeostasis of trace elements and minerals during cancer progression [44, 45], which may be related to the protective effects of the *Chromelosporium-Bradyrhizobium* cross-kingdom symbiosis in CMD.

Based on this, we hypothesize that the *Chromelosporium-Bradyrhizobium* symbiosis likely participates in the chlorophyll/heme-tryptophan metabolic network under Zacopride intervention, forming a conditional symbiotic relationship within the host intestinal microbial system. It may act as a cascade regulatory node to integrate metabolic processes and restore oxidative stress homeostasis. However, *Chromelosporium* was not identified as an important feature in the CMD-ZAC model, and no significant differential metabolic pathways were observed for fungi between groups. This suggests that it functions as a "steady-state background" for ecological restoration rather than a direct drug target. It may provide metabolic support to *JC017*, *Bradyrhizobium*, and others but cannot directly explain the significant recovery of the Chlorophyll a metabolic pathway.

### Decreased abundance of Barnesiella as a key feature of gut microbiota in CMD

Machine learning models identified *Barnesiella* as a critical feature in the CMD-NC classification model, and LEfSe analysis further confirmed it as a significantly differential microbiota in the NC group. This bacterium belongs to the *Porphyromonadaceae* family and is taxonomically classified under the *Bacteroidetes* phylum. *Barnesiella* has been recognized as a key probiotic, playing an important role in regulating gut microecological balance and systemic metabolic health. Growing evidence suggests its significant contribution to the production of SCFAs, particularly butyrate and isobutyrate, along with smaller amounts of succinate, propionate, and acetate [46]. These metabolites are crucial for maintaining intestinal barrier integrity, modulating immune responses, and influencing host metabolism. This aligns with clinical observations by Song et al., who reported lower relative abundance of *Barnesiella* in individuals with obesity and chronic inflammation, indicating its potential as a microbial biomarker or therapeutic target in metabolic diseases [47]. The negative correlation between *Barnesiella* abundance and circulating triglycerides, interleukin-1 (IL-1), and interleukin-6 (IL-6), as well as its positive correlation with the anti-inflammatory interleukin-10 (IL-10), further highlights its immunomodulatory capabilities [48]. In our study, the downregulated abundance of *Barnesiella* in the CMD group emerged as a distinctive effect of CMD on gut microbiota dysbiosis, influencing SCFAs metabolism and persisting throughout disease progression.

## Limitations

Firstly, the sample size of this study remains relatively limited, which may expose the machine learning models to the risk of overfitting, potentially leading to an overestimation of predictive performance due to small-sample bias. Secondly, the current evidence is primarily focused on the correlation between the microbiome and metabolic pathways, and has not yet crossed the threshold into causal inference. The current model validation primarily relies on functional assessment via Doppler echocardiography, without incorporating histopathological or metabolic analyses, which represents a key direction for improvement in future studies through the integration of multimodal evaluations. However, as an exploratory study, this represents a crucial first step in uncovering the role of the gut microbiota-metabolic network in the pathological progression of CMD. This study revealed the significant roles of three key microbial biomarkers and cross-kingdom bacterial-fungal symbiotic relationships, with *JC017* and the heme/chlorophyll-tryptophan metabolic network clearly standing out as the central findings. Although we have preliminarily identified the potential of *JC017* as a probiotic and biomarker for CMD and uncovered its correlation with the Chlorophyll a metabolic pathway, its specific mechanisms of action still require further in-depth exploration and validation. Our next steps will focus on the following directions: (1) establishing the causal relationship between *JC017* and the therapeutic effects of Zacopride; (2) elucidating the specific mechanisms by which *JC017* influences the heme/chlorophyll-tryptophan metabolic network through the Chlorophyll a metabolic pathway; and (3) validating the potential application value of *JC017* as a biomarker and therapeutic target in CMD.

## Conclusion

This study investigated the dynamic regulatory mechanisms of the gut microbiome in the progression of CMD and the therapeutic effects of Zacopride. It revealed that *JC017*, *Barnesiella*, and the *Chromelosporium-Bradyrhizobium* cross-kingdom symbiosis play critical roles in CMD pathology and treatment, demonstrating potential as biomarkers. The synchronized changes in *JC017* abundance and the Chlorophyll a metabolic pathway suggest that it may delay CMD progression by directly or indirectly regulating the chlorophyll/heme-tryptophan metabolic network, making it a potential target for Zacopride intervention. The synergistic interaction between *Chromelosporium* and *Bradyrhizobium* may dynamically balance oxidative stress and immune homeostasis through the chlorophyll/heme-tryptophan metabolic axis, with the disruption of their symbiotic relationship potentially providing a new microbiological explanation for CMD. Additionally, the significant reduction in *Barnesiella* abundance and its association with SCFAs synthesis and anti-inflammatory signaling pathways confirm that gut microbiota dysbiosis in CMD is linked to SCFAs-dependent immune regulation and exacerbated metabolic inflammation. However, further exploration is needed to validate the functional roles and molecular mechanisms of these microbial taxa, particularly *JC017*, as biomarkers for predicting CMD phenotypes, and to elucidate the fine-tuned regulation of the gut microbiota-chlorophyll/heme-tryptophan metabolic pathway in CMD.

## Supplementary Information

Below is the link to the electronic supplementary material.

Supplementary Material 1

Supplementary Material 2

Supplementary Material 3

Supplementary Material 4

Supplementary Material 5

Supplementary Material 6

Supplementary Material 7

Supplementary Material 8

Supplementary Material 9

Supplementary Material 10

Supplementary Material 11

## Data Availability

The data that support the findings of this study are openly available in the NCBI Sequence Read Archive (SRA) at https://www.ncbi.nlm.nih.gov/sra/PRJNA1240267, under accession number PRJNA1240267.

## Abbreviations

CMD: Coronary microvascular dysfunction
ASVs: Amplicon sequence variants
LAD: Left anterior descending coronary artery
CFR: Coronary flow reserve
RF: Random forest
LEfSe: Linear discriminant analysis effect size
TTDE: Transthoracic Doppler echocardiography
AUC: Area under the curve
MACE: Major adverse cardiovascular events
PCoA: Principal coordinate analysis
ENET: Elastic net
STZ: Streptozotocin
RPFV: Resting peak flow velocity
SPFV: Stress-induced peak flow velocity
