# Supplementary figures and images for: Effects of zacopride and multidimensional impacts of cross-kingdom symbiosis: gut microbiota modulates coronary microvascular dysfunction via the chlorophyll/heme-tryptophan metabolic axis
Source: J Transl Med. 2025 Oct 14;23:1097. doi: 10.1186/s12967-025-07048-3 (PMC12522788; doi:10.1186/s12967-025-07048-3)

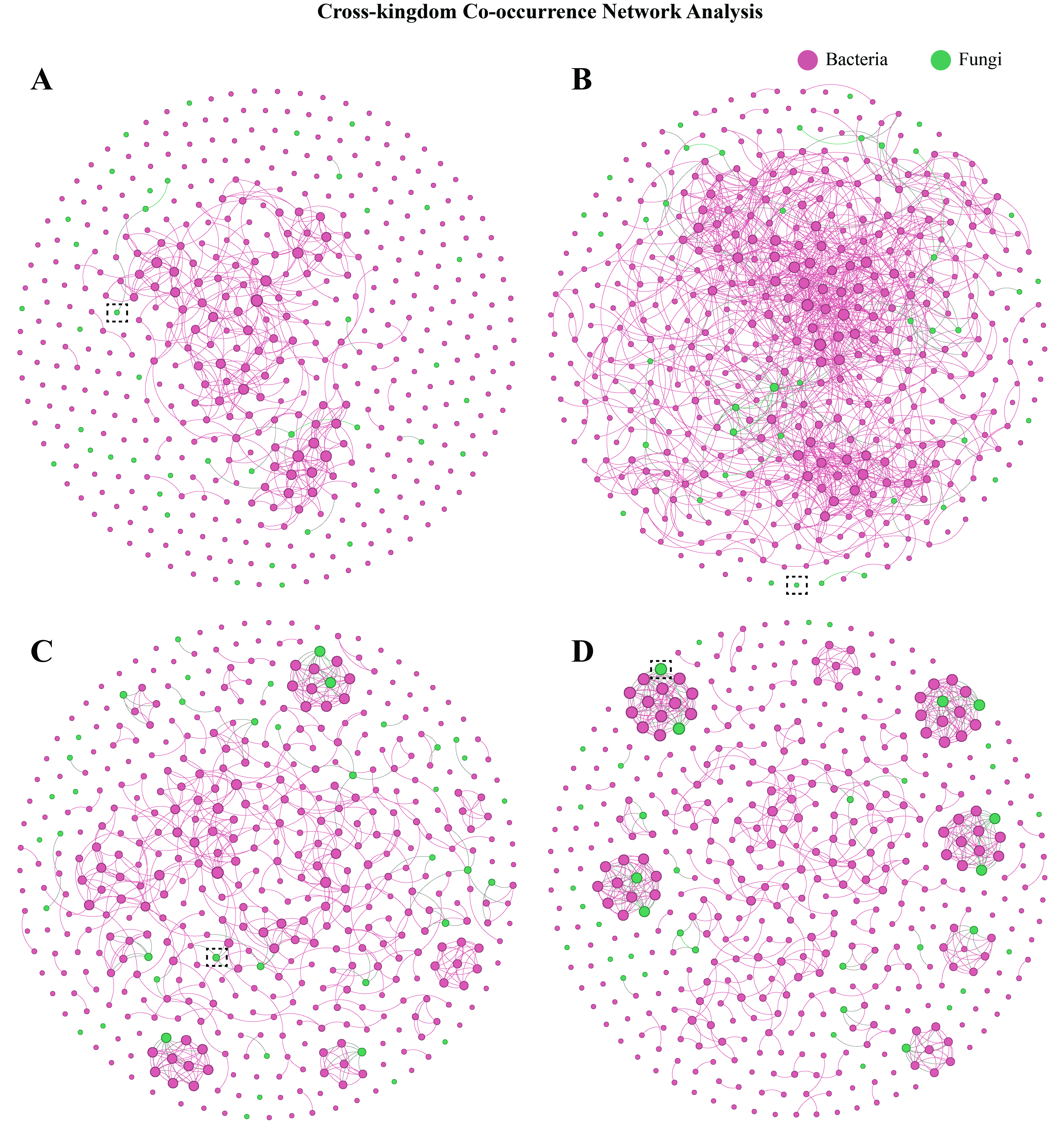

Supplement: Supplementary file 2 — Supplementary Material 2 [file 12967_2025_7048_MOESM2_ESM.tif]

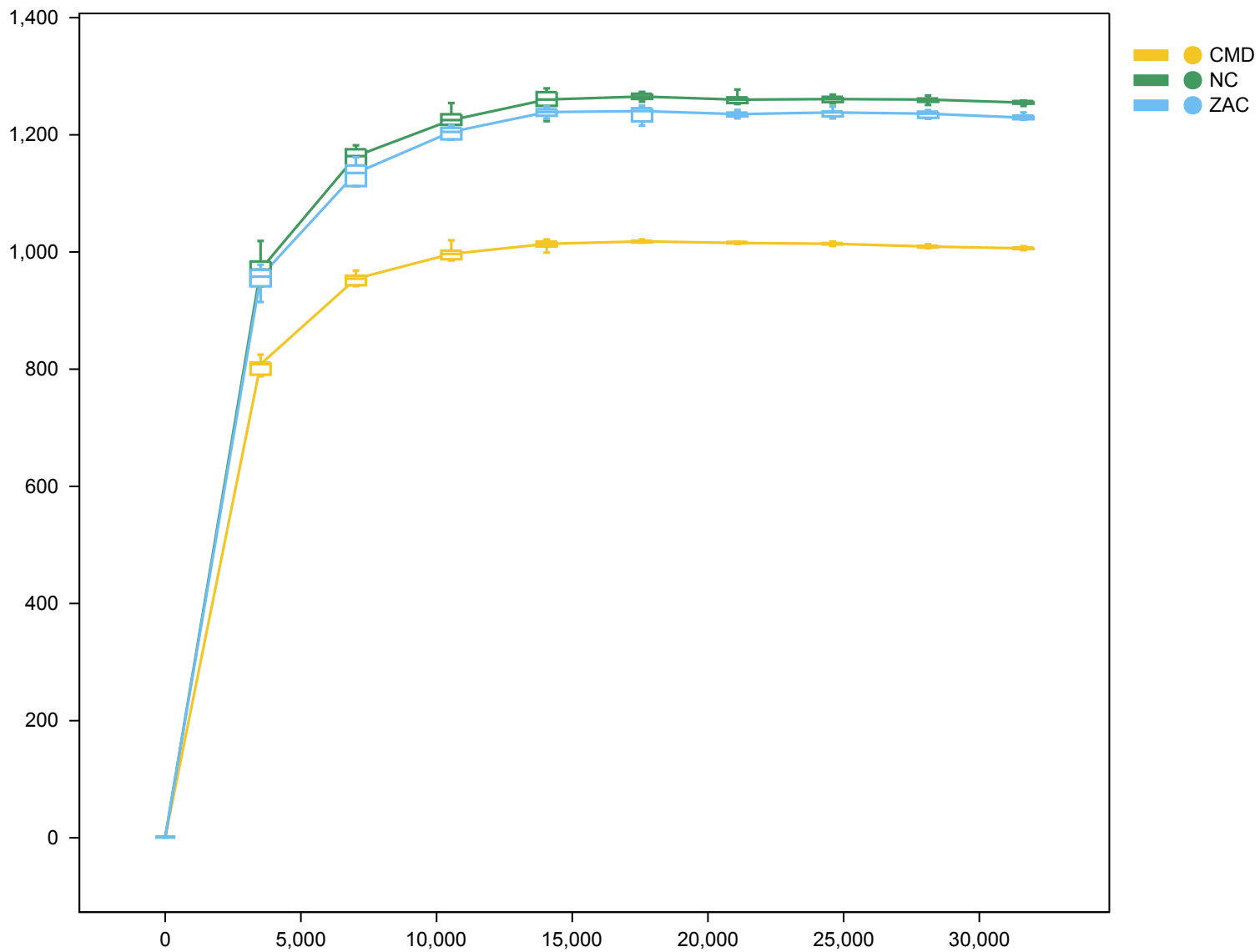

Supplement: Supplementary file 3 — Supplementary Material 3 [file 12967_2025_7048_MOESM3_ESM.pdf]

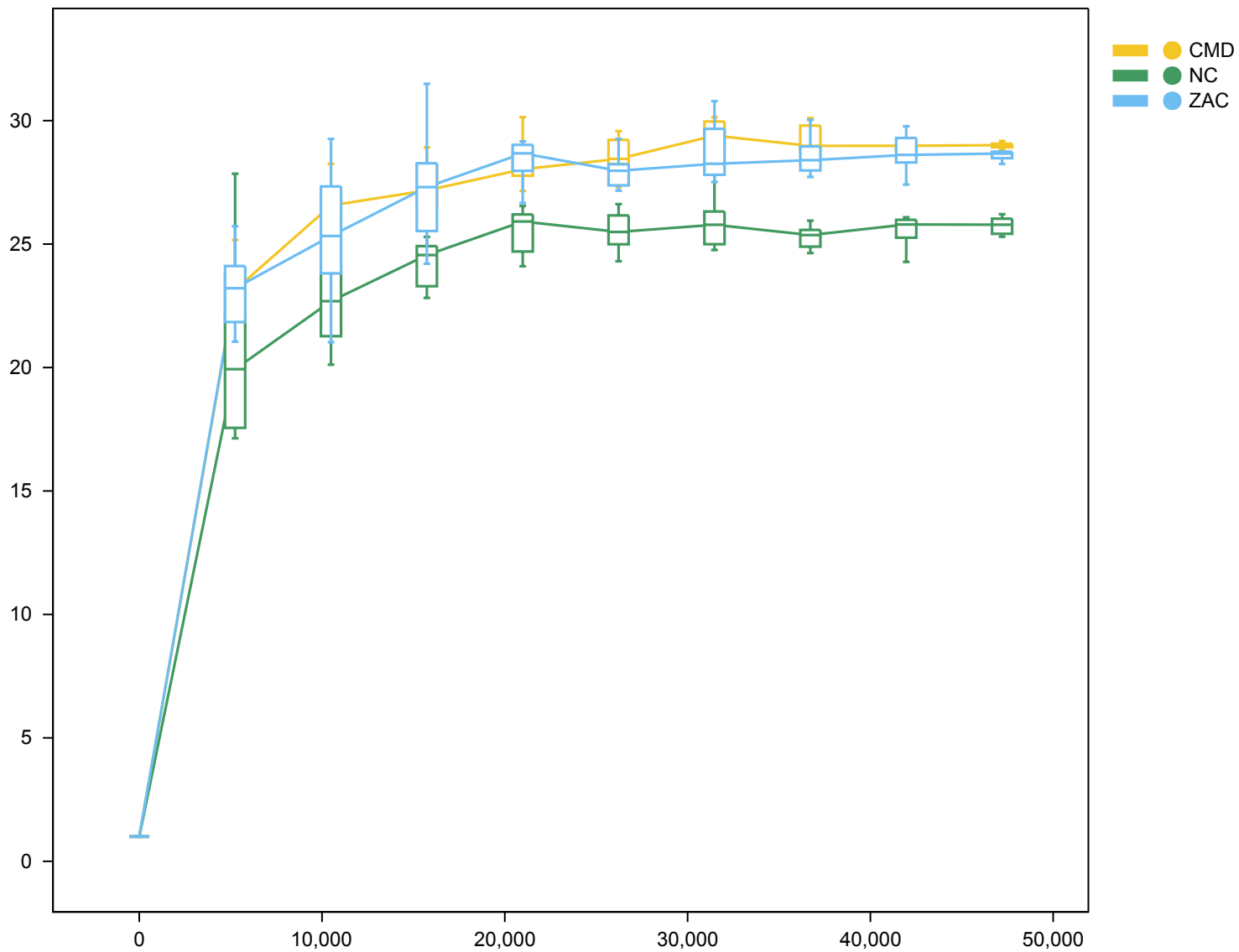

Supplement: Supplementary file 4 — Supplementary Material 4 [file 12967_2025_7048_MOESM4_ESM.pdf]

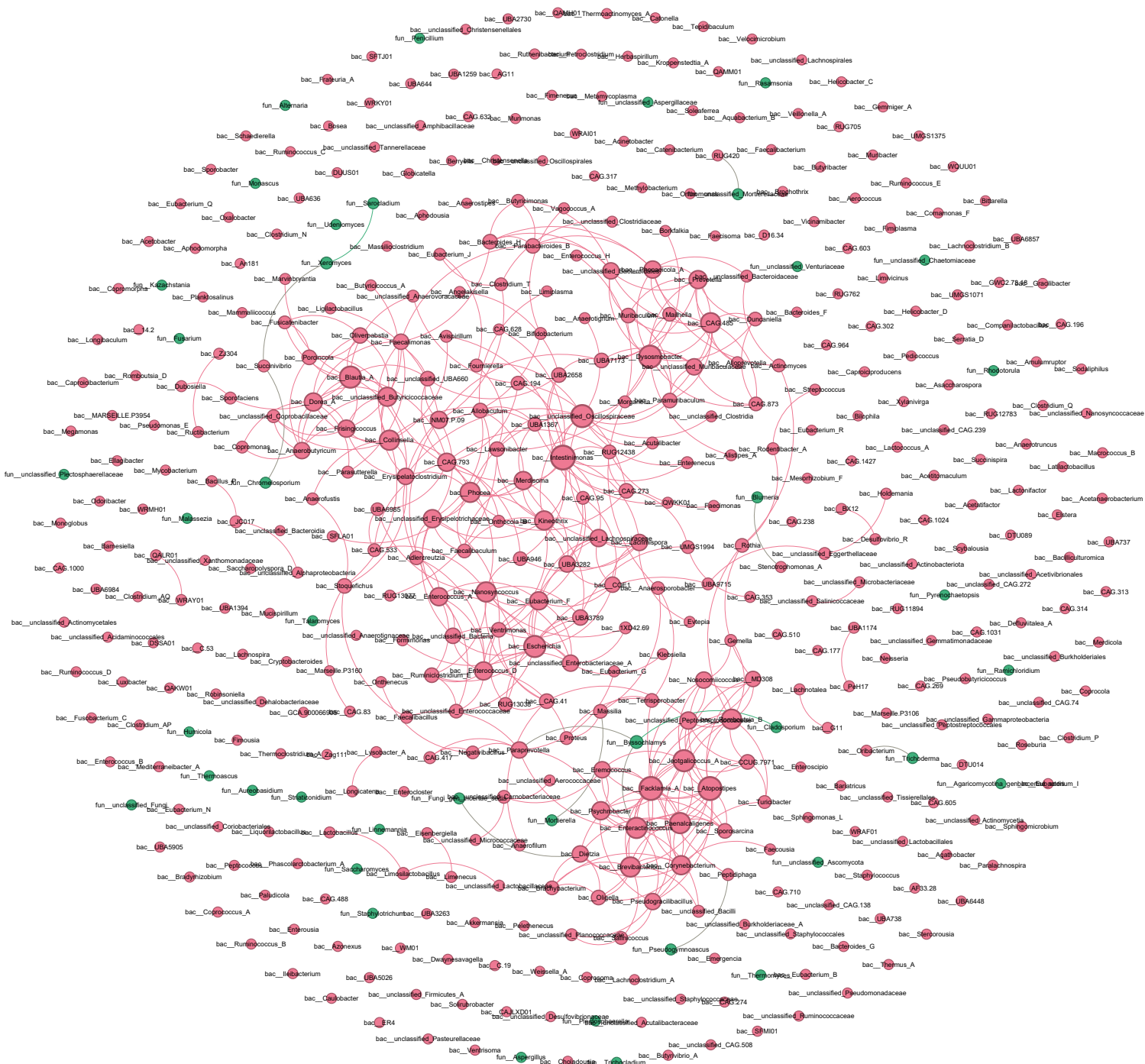

Supplement: Supplementary file 7 — Supplementary Material 7 [file 12967_2025_7048_MOESM7_ESM.pdf]

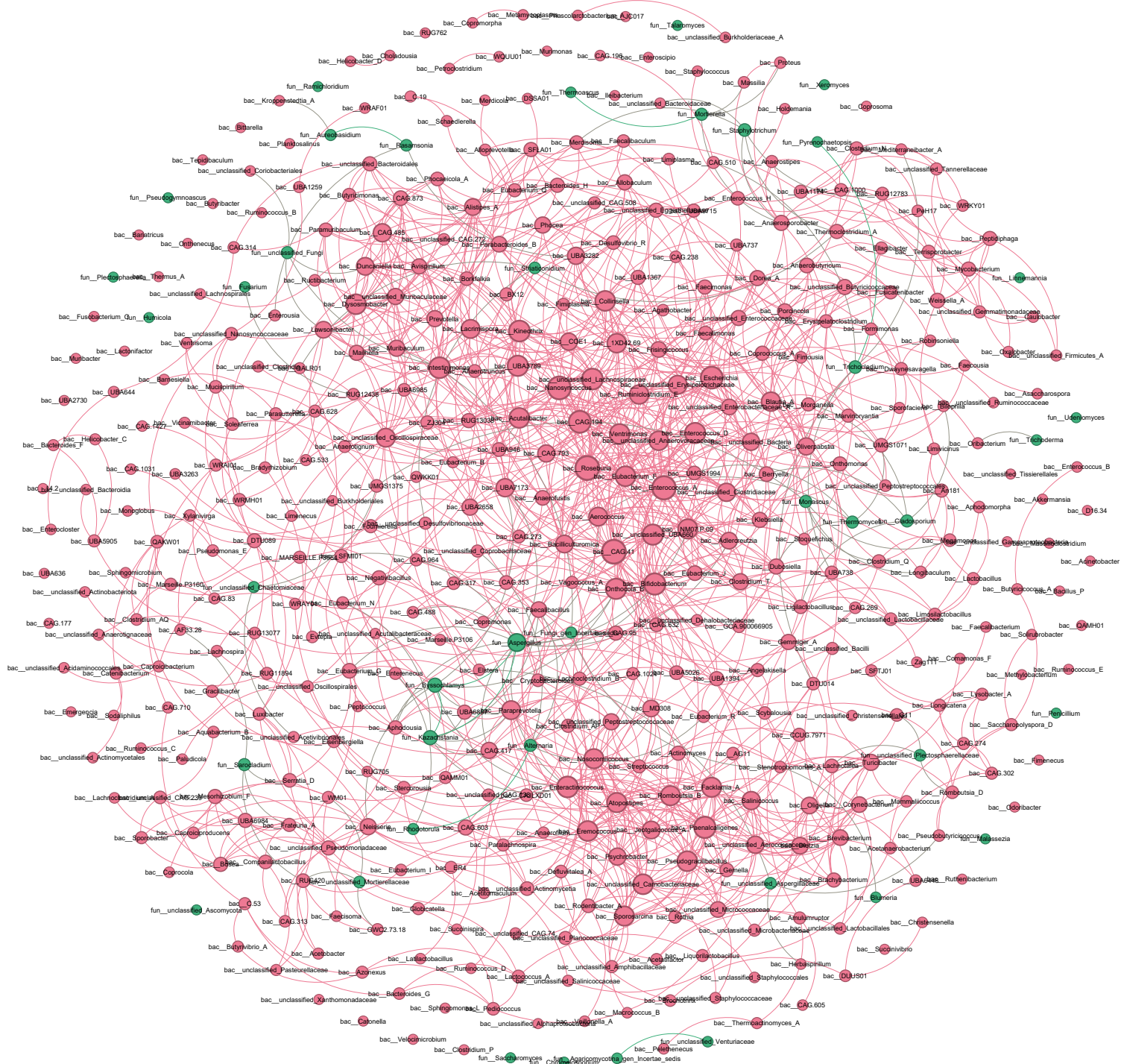

Supplement: Supplementary file 8 — Supplementary Material 8 [file 12967_2025_7048_MOESM8_ESM.pdf]

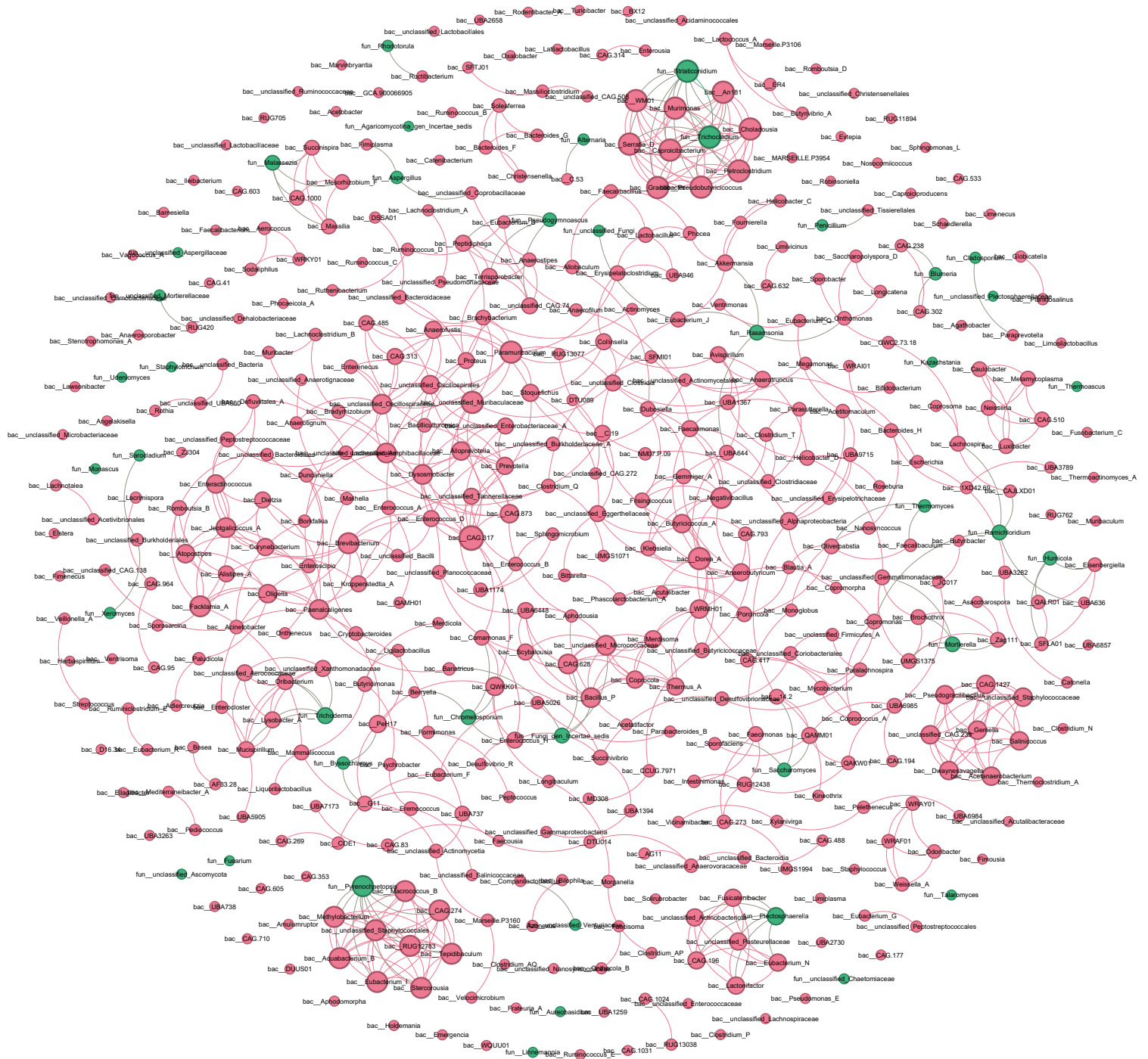

Supplement: Supplementary file 9 — Supplementary Material 9 [file 12967_2025_7048_MOESM9_ESM.pdf]

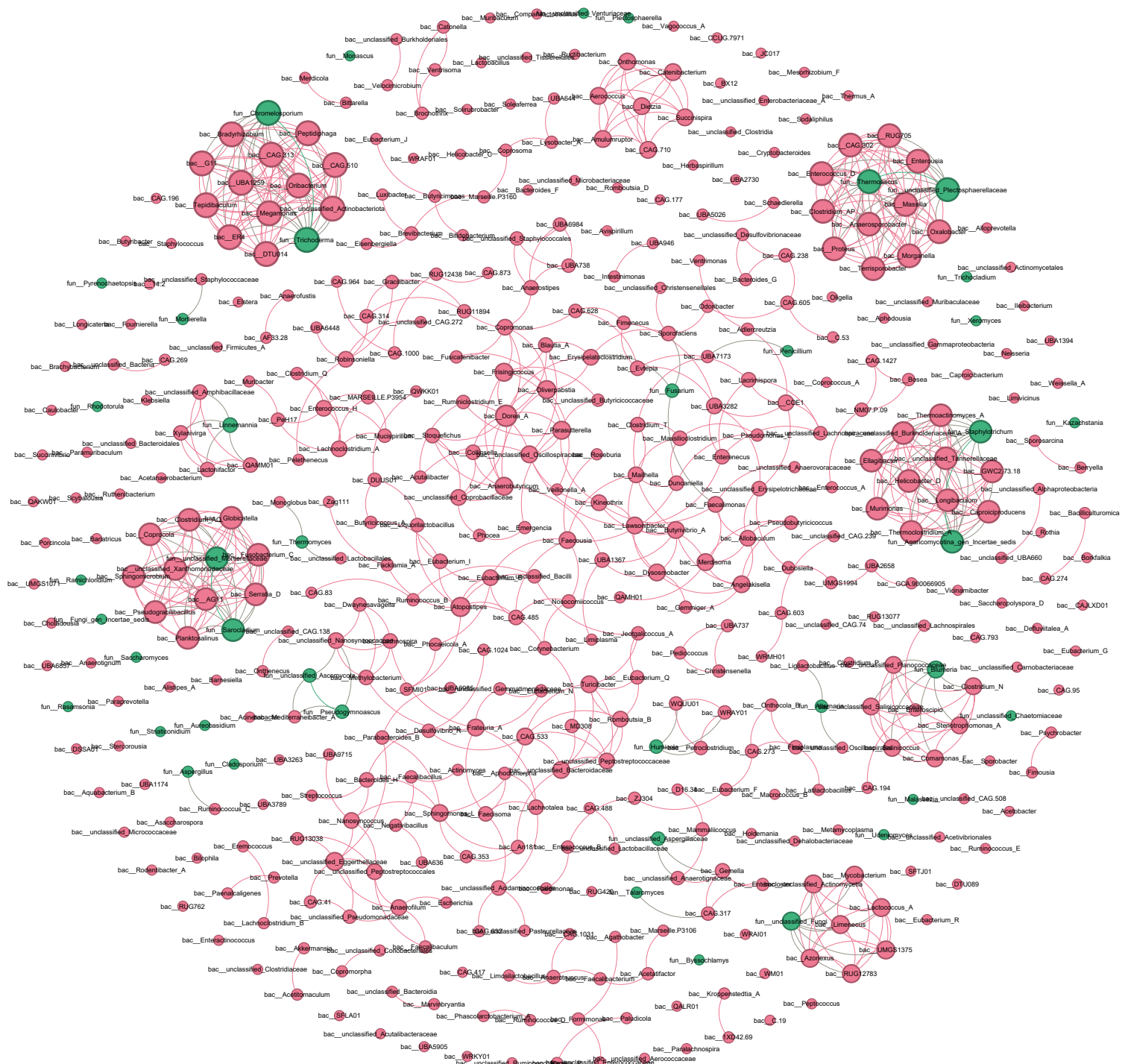

Supplement: Supplementary file 10 — Supplementary Material 10 [file 12967_2025_7048_MOESM10_ESM.pdf]
